# Adherence to Diabetes Mellitus Treatment Regimen Among Patients With Diabetes in the Tabuk Region of Saudi Arabia

**DOI:** 10.7759/cureus.30688

**Published:** 2022-10-25

**Authors:** Mansuor Alanazi, Amirah M Alatawi

**Affiliations:** 1 Family and Community Medicine, Faculty of Medicine, University of Tabuk, Tabuk, SAU

**Keywords:** tabuk region, diabetes complications, blood sugar management, patient adherence, diabetes treatment, diabetes mellitus

## Abstract

Background

Diabetes Mellitus (DM) types 1 and 2 and their complications are becoming more prevalent in Saudi Arabia. Non-adherence to diabetes management techniques could result in inadequate blood sugar management causing treatment failure, the rapid development of comorbidities, and higher mortality in patients with diabetes.

Objectives

This study investigated the adherence of patients with diabetes in the Tabuk region of Saudi Arabia to their prescribed medications and examined the association between adherence and other sociodemographic characteristics.

Methods

This cross-sectional study was conducted in Tabuk, Saudi Arabia, among patients with diabetes. A validated online self-administered questionnaire was provided to the research participants using Google Forms. Participants were selected using a convenient non-probability sampling method. The chi-square test was used to compare qualitative data whereas quantitative data were expressed as frequency and percentage.

Results

Overall, 380 participants were enrolled in this study. The study population’s average diabetes treatment adherence score was 9.6 ± 3.3% from a maximum score of 15 (range: 0-15). Overall, 293 (77.1%) participants were adherent, whereas 87 (22.9%) were non-adherent. In addition, more than one-third of the individuals did not miss medication doses or follow-up appointments. However, forgetfulness was the most common cause of missed medicine doses and follow-up appointments. In addition, several sociodemographic characteristics, including marital status, nationality, geographic region, and employment position, were signiﬁcantly associated with DM treatment adherence (*P *= 0.001, 0.002, 0.003, and 0.002, respectively).

Conclusion

Most individuals in Tabuk, Saudi Arabia, showed adequate DM medication adherence. Forgetfulness was the most common cause for missed medicine doses and follow-up appointments while several socioeconomic factors including marital status, nationality, geographic region, and occupation were associated with treatment adherence. Therefore, intervention strategies and public health campaigns should be implemented to increase treatment adherence among patients with DM.

## Introduction

Diabetes mellitus (DM) is the most prevalent chronic condition of the endocrine system, which is characterized by improper glucose metabolism in the human body. Notably, blood glucose levels increase due to a malfunction in the enzymatic activity of insulin. According to the European association, Diabetes mellitus Type 1 and Type 2 (T1DM and T2DM) are the most common subtypes of DM with over nine million people affected with T1DM and approximately 462 million people affected by T2DM [1].

Globally, the prevalence of DM is extremely high and its increased prevalence is attributed to high healthcare and economic costs. In 2013, the expected economic cost of diabetes in Saudi Arabia was $2.4 billion, which was expected to rise by another $6.5 billion in 2020 [2]. In the United States, approximately $16,750 was projected to be spent annually on medical treatment for patients with diabetes, of which $9,600 was allocated only for diabetes management [3,4].

The American Diabetes Association (ADA) has developed a standard of care for diabetes management that addresses healthcare practitioners, patients, and others who may be involved in diabetes care [5]. Notably, improved medication and treatment adherence can signiﬁcantly reduce economic and healthcare problems. For example, it was projected that a 10% improvement in diabetic treatment adherence might result in annual savings of $450 [6,7].

It is crucial to understand and assess the factors inﬂuencing patients’ adherence to diabetes treatment to reduce the economic and health burden associated with DM. Several variables such as age, sex, socioeconomic status, negative perceptions of medication, and health awareness, may inﬂuence patients’ adherence to diabetic treatment options [8]. Diabetes treatment methods along with adherence to diabetes management procedures, such as medication and lifestyle modiﬁcations, can reduce the overall healthcare burden of diabetes. A systematic analysis consisting of 29 studies found a 77% pooled uncontrolled T2DM prevalence in Saudi Arabia and the most common predictors were longer duration of diabetes, lack of diabetes awareness, and low self-efficacy [9].

Menti et al. found that Health locus of control (HLoC), self-eﬃcacy, and a more positive illness perception were signiﬁcant predictors of treatment adherence among the studied diabetes patients [10]. Previously, Kang et al. discovered cost-related medication non-adherence (CRN) in 16.5% of adult patients with diabetes in the United States. They discovered that patients with diabetes with health insurance and an annual income greater than $50,000 were less likely to report CRN [11].

Several studies on diverse demographics in the Arab region have examined the prevalence and variables inﬂuencing non-adherence to diabetic treatment [12-19]. Asheq et al. in UAE analyzed 61.67% of patients in the low adherence category [12]. Al-Qerem et al. identiﬁed 46.5% moderate and 12.2% low medication adherence due to insuﬃcient information about the medication requirement, frequency, and concerns over its dangers as the primary determinants of poor adherence in the study group [13].

Saudi Arabia is vulnerable to the global diabetes epidemic and has the second-highest rate of DM in the Middle East, with seven million and three million patients with diabetes and pre-diabetes, respectively [14]. However, few studies have investigated the treatment adherence and compliance levels among patients with diabetes residing in various regions of Saudi Arabia. Ahmed et al. conducted a study and the results revealed that 45.5% of patients with diabetes skipped their follow-up appointments and 54.8% of the patients were non-compliant with follow-up schedules, medication instructions, and healthy food suggestions [15].

In previous studies conducted in the Al Hasa district [16], Bisha Governorate [17] and Khobar city [18], the non-adherence prevalence to diabetic therapy was 67.9%, 21.4% (n = 375), and 64.2% (n = 212), respectively, among patients with diabetes [16-18]. Recently, a systematic review of the literature showed that the major factors associated with non-adherence to diabetic treatment among patients in Saudi Arabia include forgetting the medication dose, complexity due to multiple drugs and regimens, experiencing medication side effects, belief of medication ineffectiveness or less effectiveness, and discontinuing medication due to perceived improved health [19].

The ill effects include both micro and macro complications as a result of non-adherence to diabetes treatment, including retinopathy, nephropathy, neuropathy, inflammation and oxidative stress, a diabetic foot that may lead to amputation, gum diseases and other oral diseases as well as cancer if not prevented and managed in time [20]. Diabetes treatment strategies cannot produce successful health outcomes if adherence to the treatment plan is not ensured. Patients’ adherence to diabetes management protocols, including medication and lifestyle interventions, may help in reducing the overall healthcare burden of diabetes.

Further, it is important to understand and evaluate the elements involved in patients’ adherence to diabetes treatment in order to reduce the economic and health burden associated with diabetes mellitus. This understanding is especially required for the development of effectively tailored treatment strategies for patients. Several factors such as age, gender, socioeconomic background, negative perception about medication, and health literacy of patients may affect the level of adherence to diabetic treatment strategies [8]. Therefore, it is important to carry out more studies in Saudi Arabia that evaluate underlying factors creating hindrances in the adherence of patients to treatment guidelines.

This study aimed to determine the level of treatment adherence among patients with diabetes, living in the Tabuk region of Saudi Arabia, as non-compliance increases the risk for the development of complications. Several studies have been conducted in Saudi Arabia to assess the level of treatment adherence. However, no studies have been conducted in the Tabuk region, which is the main purpose of conducting this study. Further, this study also examined the association between adherence and other sociodemographic characteristics to assess if demographic variables do interfere with the level of treatment adherence among patients with diabetes.

## Materials and methods

A cross-sectional study was conducted in Tabuk, Saudi Arabia from April 2022 to June 2022. The research population was recruited from the general diabetes population. The inclusion criteria were any adult, diabetic, on treatment, Tabouk resident of any nationality, both genders, who agreed to participate in the study, could read, and had a social media account. While the non-diabetic, diabetic without treatment, non-Tabuk residents, and refusal to participate in the study were excluded from the study population.

The sample size was determined using the Epi information tool (statistical tool for epidemiology information) based on a 95% conﬁdence interval, an error margin of 5%, and the total population in Tabuk, Saudi Arabia. The estimated sample size of 384 participants was increased to 422 to account for the 2010% non-response rate.

The study was conducted using an online self-administered pre-tested questionnaire translated into Arabic and administered through Google Forms. The generated link was shared randomly on social media platforms, such as Facebook, WhatsApp, Telegram, and Twitter. The interface communicated the purpose of the investigation. A convenient nonprobability sampling method was used to acquire data from the participants.

A validated questionnaire was used based on previous research [17,18,21,22]. In addition, the questionnaire covered sociodemographic information about the participants, including age, gender, nationality, and place of residence. Furthermore, the questionnaire contained questions about adherence to diabetes treatment among patients with diabetes in Tabuk, Saudi Arabia, including questions about the type of DM, medications used by the patient, adherence, and factors inﬂuencing adherence to DM medications.

The questionnaire was pretested on a sample of 20 participants in a pilot study whose results were not included in the main study. Modiﬁcations were made to ensure clarity and a simple understanding of the questions. Data were coded, processed, and analyzed using the Statistical Package for the Social Sciences (SPSS) version 23.0 (IBM Corp., Armonk, NY). Quantitative data were expressed as numbers (N) and percentages (%). The chi-squared test was used to compare qualitative data between the two groups.

The Tabuk University Research Ethics Committee approved the study (approval No UT-192-48-2022 on 2022-04-11). All information was downloaded as an excel (EXCL) sheet, kept conﬁdential by the primary author, and used solely for research purposes before being discarded. All participants provided informed consent before completing the questionnaire.

## Results

Females comprised 50.8% of the 380 participants in our study, while 49.2% were males. One-third of the participants were in the age group of 31-40 (37.2%), while participants older than 56 constituted the smallest proportion of the study group. Regarding marital status, most participants were married (63.7%), and most study participants were Saudi Arabians (88.7%). Our ﬁndings revealed that 43.2% of the participants completed their secondary education, and approximately the same proportion earned a bachelor’s degree. According to the geographic distribution of participants, the majority (36.6%) were from the western region, followed by participants from the northern (20.5%), southern (16.8%), eastern (15%), and middle (10.8%) regions. Less than 50% of individuals were employed, 17.1% were unemployed, and 6.3% were retired. Furthermore, concerning the average monthly income (in Saudi Riyal {SAR}) of our participants, the majority earned between 10,000 and 14,999 SAR (Table 1).

**Table 1:** Socio-demographic characteristics of the study participants (n=380) SAR: Saudi Riyal

Variable	Category	Frequency	Percent
Gender	Male	187	49.2%
	Female	193	50.8%
Age (years)	18-30	118	31.1%
	31-40	142	37.4%
	41-55	83	20.8%
	56-70	29	7.6%
	> 70	8	2.1%
Marital status	Single	61	16.1%
	Married	242	63.7%
	Divorced	57	15%
	Widowed	20	5.3%
Nationality	Saudi	337	88.7%
	Non-Saudi	43	11.3%
Education	Primary	7	1.8%
	Intermediate	19	5%
	Higher secondary	164	43.2%
	Bachelor	164	43.2%
	Other	26	6.8%
Region	Northern	78	20.5%
	Southern	64	16.8%
	Middle	41	10.8%
	Eastern	57	15%
	Western	140	36.8%
Occupational status	Employed	182	47.9%
	Unemployed	65	17.1%
	Private business	89	23.4%
	Retired	24	6.3%
	Other	20	5.3%
Average monthly income (SAR)	< 10,000	149	39.2%
	10,000 - 14,999	177	46.6%
	15,000 - 20,000	43	11.3%
	> 20,000	11	2.9%

**Figure 1:**
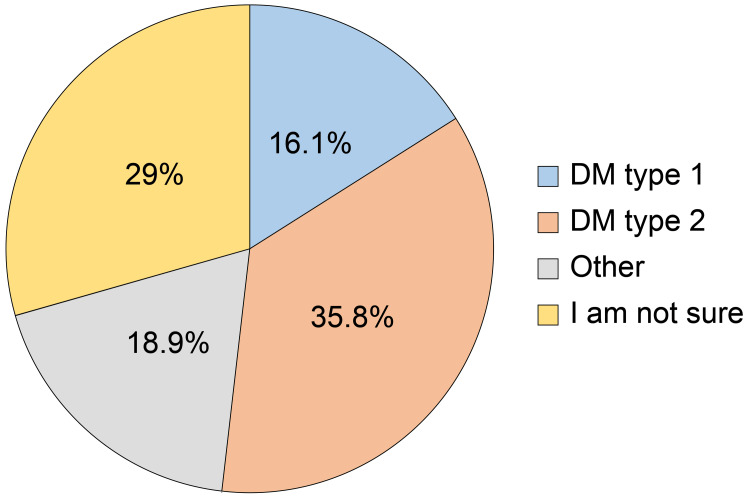
What is the type of diabetes mellitus (DM) you are affected with?

According to our ﬁndings, the average adherence score for the study population was 9.6 ± 3.3% from a maximum of 15 (range: 0-15). Overall, 293 participants (77.1%) were adherent, whereas 87 (22.9%) were non-adherent. Only 16.1% of the participants were diagnosed with T1DM, making T2DM the most common type of diabetes among the participants. Nevertheless, 29% of our participants were uncertain about their type of diabetes (Figure 1).

Our ﬁndings revealed that 45.8% of participants had no family history of diabetes, 42.4% admitted having a family history of diabetes, and 11.8% were unsure. In addition, over 60% of participants indicated that they regularly monitor their blood sugar levels, whereas 29.5% did not. More than 50% of responders typically had normal blood glucose levels. In addition, we found that 42.1% of participants had comorbidities combined with DM, whereas approximately 33% did not. Unfortunately, more than half of the participants acknowledged altering their prescription dosages independently. Furthermore, we discovered that 40.3% of participants considered DM treatment a hardship or inconvenience. Table 2 shows that 28.2% felt fatigued and considered discontinuing diabetic treatment, whereas the majority did not (64.7%).

**Table 2:** History of diabetes and treatment adherence

Question	Yes	No	Not sure
Do you have a family history of DM?	161 (42.4%)	174 (45.8%)	45 (11.8%)
Do you regularly monitor your blood sugar level?	226 (59.5%)	112 (29.5%)	42 (11.1%)
Does your blood glucose level remain within normal limits most of the time?	214 (56.3%)	130 (34.2%)	36 (9.5%)
Do you also have any other diseases along with diabetes?	160 (42.1%)	171 (45%)	49 (12.9%)
Do you alter your medication dose by yourself, for example, double/skip dose in case of very high/low blood glucose level?	204 (53.7%)	126 (33.2%)	50 (13.2%)
Do you often think that diabetic treatment is a burden or inconvenient for you?	153 (40.3%)	195 (51.3%)	32 (8.4%)
Do you ever feel tired and think about the discontinuation of diabetic treatment?	107 (28.2%)	246 (64.7%)	27 (7.1%)

**Figure 2:**
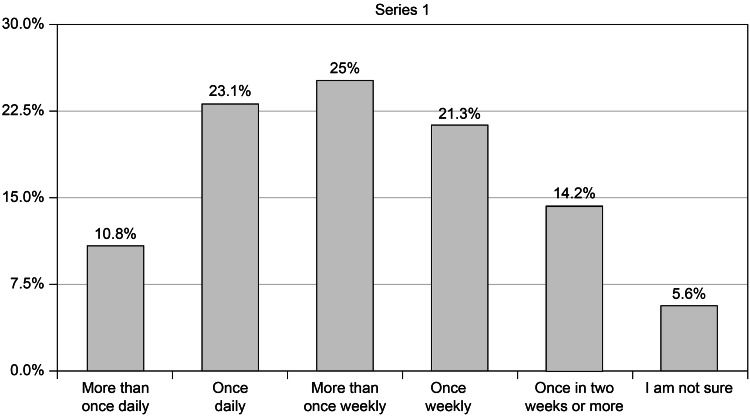
How often do you monitor your blood glucose level? (n=268)

Figure 2 presents the results regarding the frequency of blood glucose monitoring. The highest proportion of participants (25%) measured their blood glucose more than once weekly followed by 23.1%, 21.3%, 10.8%, and 14.2% who measured their blood glucose level once daily, once weekly, once bi-weeks or more, and more than once daily, respectively.

**Figure 3:**
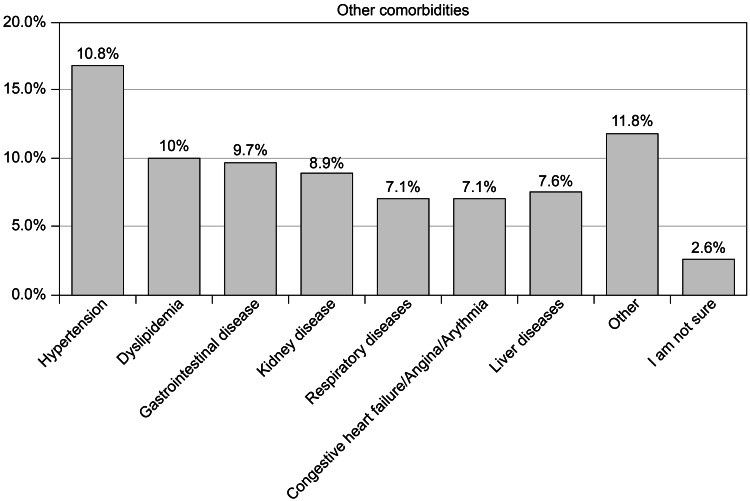
Other comorbidities among the study participants

Hypertension (16.8%) was the most commonly reported comorbidity, followed by dyslipidemia (10%), gastrointestinal disease (9.7%), renal disease (8.9%), liver disease (7.6%), respiratory disease (7.1%), and cardiac disease (7.1%) (Figure 3).

Most participants (44.5%) were prescribed two to three drugs, 24.2% were prescribed four to ﬁve drugs, and 14.1% were prescribed only one drug for the treatment of DM. Similarly, the highest proportion of responders taking overall daily medications along with anti-diabetes medications used two to three and four to ﬁve pills, respectively. Regarding participants’ knowledge of their medicines, we discovered that most participants knew some of their drugs’ names, formulas, and purposes/uses (Table 3).

**Table 3:** Drugs/regimens for the treatment of diabetes

Question	N	%
How many drugs/regimens you have been advised for the treatment of diabetes? (1, 2-3, 4-5, More than 5, I am not sure)	56, 169, 92, 33, 30	14.7%, 44.5%, 24.2%, 8.7%, 7.9%
What is the number of all drugs you take daily? (1, 2-3, 4-5, 6-8, More than 8, I am not sure)	50, 112, 112, 48, 37, 21	13.2%, 29.5%, 29.5%, 12.6%, 9.7%, 5.5%
What is the number of anti-diabetic drugs you take daily? (1, 2-3, 4-5, More than 5, I am not sure)	96, 130, 83, 43, 28	25.3%, 34.2%, 21.8%, 11.3%, 7.4%
Do you understand the names/formula and purpose/usage of the drugs you are prescribed? (Yes, most of them; Yes, some of them; No; I am not sure)	123, 173, 52, 32	32.4%, 45.5%, 13.7%, 8.4%

**Figure 4:**
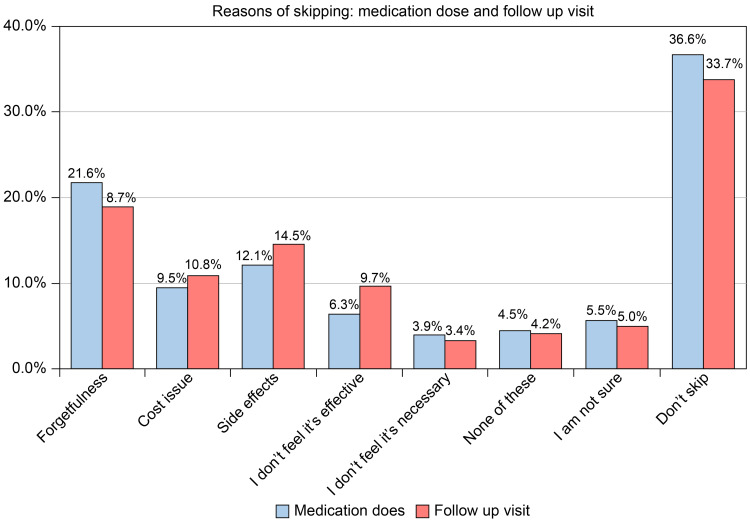
What is the reason for skipping a medication dose or follow-up visit?

In addition, our data revealed that greater than one-third of participants did not miss medication doses or follow-up appointments. However, forgetfulness was the most frequently cited cause for not taking medications as prescribed, followed by side effects and cost concerns (Figure 4).

**Figure 5:**
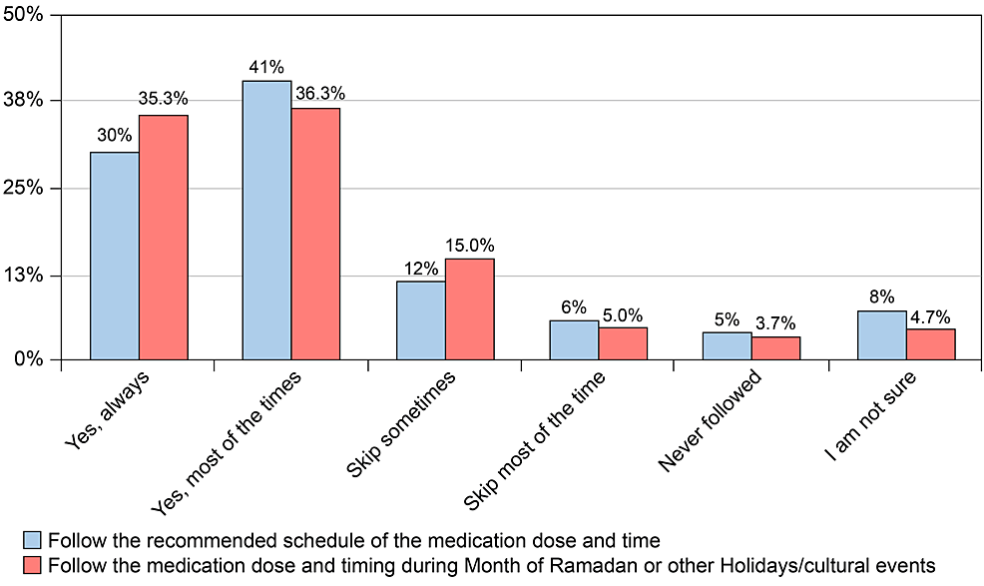
Do you follow the recommended schedule of the medication dose and time usually and during the month of Ramadan or other holidays/cultural events?

When adhering to the medication, specifically its dose and schedule were assessed on normal days and during the month of Ramadan or other holidays/cultural events, it was found that the highest proportion of participants followed the recommended schedule of the medication most of the time both in normal days (41%) and during the month of Ramadan or other holidays/cultural events (36.3%) (Figure 5).

**Figure 6:**
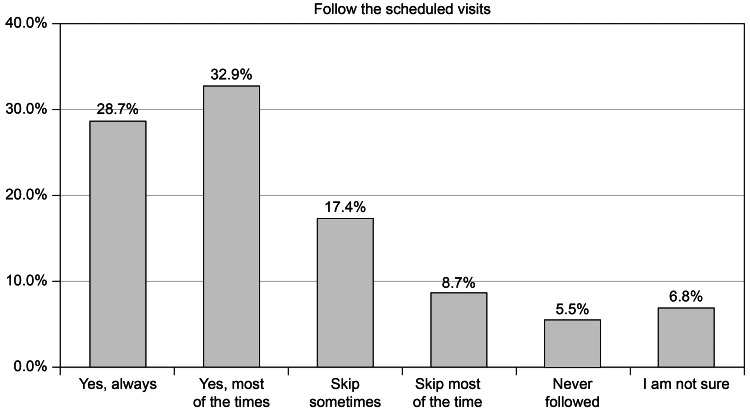
Do you always follow the scheduled visits with your doctor?

According to our data, almost one-third of the research population adhered to the regularly scheduled doctor visits. Overall, 28.7% of participants indicated that they consistently adhered to scheduled visits. However, only 5.5% of patients never adhered to their planned doctor’s appointments (Figure 6).

**Figure 7:**
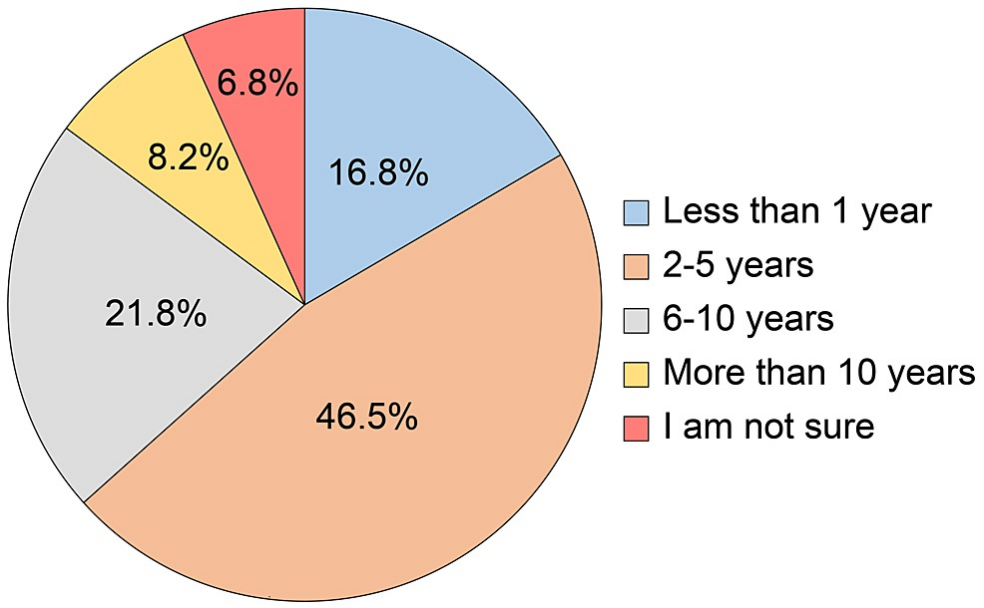
How long have you been treated for diabetes mellitus?

Regarding the duration of therapy for DM, the majority of participants (46.6%) had been treated for 2-5 years, 21.8% for 6-10 years, 16.8% for less than 1 year, and 8.2% for more than 10 years (Figure 7).

Factors associated with adherence to diabetes treatment among patients with diabetes: A signiﬁcant association between adherence to diabetes treatment and marital status, nationality, geographic location, and occupation was observed (P = 0.001 & 0.030, 0.002 & 0.008, 0.003 & 0.041, and 0.002 & 0.001, respectively). Other sociodemographic factors were not signiﬁcantly associated with adherence to diabetes treatment (Table 4).

**Table 4:** Factors associated with adherence to diabetes treatment among diabetic patients SAR: Saudi Riyal

Variable	Category	Adherence to treatment		P value	Adjusted model value
		Adherent	Not adherent		
Gender	Male	138 (73.8%)	49 (26.2%)	0.131	0.066
	Female	155 (80.3%)	38 (19.7%)		
Age (years)	18-30	92 (78%)	26 (22%)	0.467	0.396
	31-40	106 (74.6%)	36 (25.4%)		
	41-55	69 (83.1%)	14 (16.9%)		
	56-70	21 (72.4%)	8 (27.6%)		
	> 70	5 (62.5%)	3 (37.5%)		
Marital status	Single	30 (49.2%)	38 (50.8%)	< 0.001	0.030
	Married	206 (85.1%)	37 (14.9%)		
	Divorced	42 (73.7%)	15 (26.3%)		
	Widowed	15 (75%)	5 (25%)		
Nationality	Saudi	268 (9.5%)	69 (20.5%)	0.002	0.008
	Non-Saudi	25 (58.1%)	18 (41.9%)		
Education	Primary	3 (42.9%)	4 (57.1%)	0.099	0.115
	Intermediate	12 (63.2%)	7 (36.8%)		
	Higher secondary	126 (76.8%)	38 (23.2%)		
	Bachelor	132 (80.5%)	32 (19.5%)		
	Other	20 (76.9%)	6 (23.1%)		
Region	Northern	53 (67.9%)	25 (32.1%)	0.003	0.041
	Southern	49 (76.6%)	15 (23.4%)		
	Middle	33 (80.5%)	8 (19.5%)		
	Eastern	37 (64.9%)	20 (35.1%)		
	Western	121 (86.4%)	19 (13.6%)		
Occupational status	Employed	156 (85.7%)	26 (14.3%)	0.002	0.001
	Unemployed	45 (69.2%)	20 (30.8%)		
	Private business	64 (71.9%)	25 (28.1%)		
	Retired	17 (70.8%)	7 (29.2%)		
	Other	11 (55%)	9 (45%)		
Average monthly income (SAR)	< 10,000	110 (73.8%)	39 (26.2%)	0.130	0.933
	10,000 - 14,999	145 (81.9%)	32 (18.1%)		
	15,000 - 20,000	29 (67.4%)	14 (32.6%)		
	> 20,000	9 (81.8%)	2 (18.2%)		

## Discussion

This study aimed to determine the level of adherence to diabetes medication among Tabuk, Saudi Arabian residents. Diabetes control critically depends on adherence to medical recommendations [16]. However, measuring patients’ compliance with diabetes treatment is diﬃcult [23]. Medication adherence is an essential component of healthcare quality. The World Health Organization deﬁnes adherence as the degree to which an individual’s behavior, such as taking medication, following a prescribed diet, and/or implementing lifestyle modiﬁcations, aligns with the agreed-upon healthcare professional recommendations [24].

The study population’s average diabetes treatment adherence score was 9.6 ± 3.3%, from a maximum score of 15 (range: 0-15). Overall, 293 participants (77.1%) were adherent, whereas 87 (22.9%) were non-adherent. A previous study in Tabuk investigated the rate of adherence to treatment for chronic diseases, such as diabetes and hypertension, and found that it was low.

Among the participants, 76.44% adhered to their medication, while nearly 25% were non-adherent. This trend is similar to the ﬁndings of a study conducted by Prabahar et al. [25]. Compared to a study conducted in Al Hasa, Saudi Arabia, which reported a 67.9% frequency of therapeutic non-compliance among participants, our study demonstrated greater adherence to DM therapy [16]. In addition, a previous study in Saudi Arabia found a lower degree of adherence than ours; overall, 54.8%, 34.5%, and 10.7% of patients had low, medium, and good adherence degrees, respectively [15]. Similarly, a Saudi Arabian study found that only one-third of the participants had a high DM adherence degree [18]. Nonetheless, a different study conducted in the Bisha governorate of Saudi Arabia revealed an overall higher degree of adherence; overall, 35.7%, 42.9%, and 21.4% of patients reported high Morisky Green Levine Medication Adherence Scale (MGLS) (score 0), intermediate (MGLS score 1 or 2), and low (MGLS score 3) adherence degrees, respectively [16]. Another study in Uganda revealed a higher rate of DM treatment adherence (83%) [26]. This disparity in the adherence level outcomes may be attributed to the differences in awareness of the importance of medication adherence, measures to enhance adherence in different nations, and scales used to measure adherence levels in those studies.

Approximately 60% of participants indicated that they regularly monitored their blood sugar levels. This control was greater than that reported in a previous study [27]. Consistent with our ﬁndings, a study conducted in Ethiopia discovered that hypertension (61.2%) and obesity (10.8%) were the most prevalent comorbidities among the evaluated patients [27]. This is consistent with the well-established knowledge of metabolic syndrome, which substantially correlates with cerebrovascular disease in Type 2 diabetes [28,29].

The most common reason for the non-adherence to medications as prescribed was forgetfulness, followed by side effects of the medication and expenses. According to a previous study, insuﬃcient ﬁnances (37.1%) and reported side effects of treatment (29.2%) were the most frequent reasons for non-adherence [18]. Additionally, marital status, nationality, geographic region, and occupation were signiﬁcantly associated with DM treatment adherence. Nonetheless, several studies in Saudi Arabia have proven that age and sex are substantially associated with DM medication adherence [16,18]. Consistent with our ﬁndings, a second study found a statistically signiﬁcant difference between occupational position and sex [18].

This study has limitations. First, due to the adherence data dependence on participant recall, the actual prevalence of adherence may be lower than that observed in this study. Patients may experience diﬃculty recalling their routines and medication-taking behaviors, although this effect was mitigated by asking participants to recall within the preceding two weeks. Second, there was the possibility of selection bias. Moreover, self-reported data on diabetes was also a major limitation of the study. Finally, a causal relationship could not be established since this was an observational study and used only a single statistical tool that is chi-square to assess the association. To confirm the tool's findings, it is recommended to use a combination of other methods of evaluating diabetes medication adherence in the future. Furthermore, future studies should be conducted to consider these factors and other aspects that may influence adherence, such as patient-provider contact.

## Conclusions

According to our findings, most individuals in Tabuk, Saudi Arabia, showed adequate DM medication adherence. Forgetfulness and several socioeconomic factors such as marital status, nationality, geographic region, and occupation were common factors for medicine non-adherence. Therefore, intervention strategies and public health campaigns should be implemented to increase awareness among patients. Moreover, policy makers are advised to make policies that are in favor of more treatment adherence of the patients to avoid complications and their causative ill effects, thereby decreasing the economic burden on the country.
